# Supplementary material for: Unveiling the digital revolution: Catalyzing total factor productivity in agriculture
Source: PLoS One. 2025 Mar 6;20(3):e0318333. doi: 10.1371/journal.pone.0318333 (PMC11884720; doi:10.1371/journal.pone.0318333)
Supplement: Attachment — Submitted filename: Comments.docx [file pone.0318333.s001.docx]

Comments

Thank you for allowing me to review this paper.

Overall, this is an interesting paper, addressing unveiling the Digital Revolution: Catalyzing Total Factor Productivity in Agriculture in China.

The article has a potential for publication, but in my view, the current version needs some revision to enhance its empirical contribution.

The paper must make an effort on the formatting. Please make a stronger effort in this regard.

No references were cited in the introduction section. Authors must provide the references.

Review the formatting in the literature review section line 3: services(Crawford , 2018); ligne 7: post-2012(Xu and Zhang, 2020) et line 10: context(Zhao *et al.*, 2020); line 15 below(Bo and Zhang, 2021); line 22: enterprises(Chen and Hu, 2022); line 28: productivity(Gao, 2015); line 34: productivity(Liu *et al.*, 2019); line 38: provinces(Li and Li, 2020); line 43: productivity landscape(Gong *et al.*, 2020); line 53: agriculture(Wang *et al.*, 2020); line 55: regions(Xue *et al.*, 2020). Line 62: efficiency(Xia *et al.*,

2019).

Line 41 what does this mean productivity[10]? Is it a reference?

The formatting should be checked throughout the document.

Section hypothesis line 12: As China's agricultural development encounters bottlenecks, The simple infusion of factors alone is insufficient to meet the growing demand for high-quality agricultural products

**Methodology**

Suggestion: Authors should describe all variables used in regression analyses and their sources. They should also describe whether the variables used are ordinal, binary, or other.

The results are very interesting but lack a discussion with other work in the same field in China and elsewhere.

It will also be useful to specify the corresponding number (N) for each region in Table 6.

Reference:
