# Supplementary material for: Unveiling the digital revolution: Catalyzing total factor productivity in agriculture
Source: PLoS One. 2025 Mar 6;20(3):e0318333. doi: 10.1371/journal.pone.0318333 (PMC11884720; doi:10.1371/journal.pone.0318333)
Supplement: Attachment — Submitted filename: Response to reviewers.docx [file pone.0318333.s002.docx]

**Revision notes**

Dear editors and reviewers,

We sincerely thank you for your feedback on our manuscript, “Unveiling the digital revolution: Catalyzing total factor productivity in agriculture”. Below, we provide a detailed revision note addressing the comments raised by the reviewers and the editorial team.

In this document, we present the reviewers’ and editors’ comments in black text, followed by our responses and explanations in blue. Thank you for your thoughtful suggestions, which have significantly improved our work.

1. The measurement of the dependent variable, TFP, is crucial to the study, yet it lacks sufficient explanation. The authors use Stochastic Frontier Analysis (SFA) with only two inputs: the number of agricultural workers and fixed capital stock. This approach may miss critical inputs such as land, water usage, and energy, which are particularly important in agriculture. The paper should provide more justification for using SFA and discuss its strengths and limitations. It should also explain why alternative methods such as Data Envelopment Analysis (DEA) or Growth Accounting were not considered. This would strengthen the credibility of the TFP measure.

Author's response: We sincerely thank the reviewer for highlighting the issues in our calculation of agricultural TFP. Upon reflection, we recognize that our initial approach lacked thorough consideration of agriculture-specific factors, such as land and agricultural machinery, which can significantly influence TFP. Additionally, our choice of methodology was not sufficiently comprehensive. Specifically, while the SFA method can estimate the rate of change in agricultural TFP, it does not directly provide the absolute level of TFP.

To address this issue, we made the following revisions: First, in calculating agricultural TFP, we drew on the methodologies of Lin(1992) and Gong(2018). Specifically, we selected input and output indicators as follows: the output indicator is defined as the total gross output value of agriculture, forestry, animal husbandry, and fisheries in each region (in million RMB). This value was adjusted to constant prices using the agriculture, forestry, animal husbandry, and fisheries GDP index with 2011 as the base year. The input indicators include total mechanical power (in 10,000 kW), fertilizer usage (in 10,000 tons), rural labor employment (in 10,000 people), and total sown crop area (in 1,000 hectares).

Second, regarding the calculation method, we note that both the DEA and SFA approaches can only provide the contemporaneous rate of change in TFP, rather than its absolute level. Since this study focuses on the impact of the digital economy on the level of agricultural total factor productivity (TFP), we adopted the classic Solow residual method and the fixed-effects method to measure agricultural TFP, denoted as TFP_OLS and TFP_FE, respectively. To minimize the influence of time-specific factors, we also controlled for time fixed effects during the estimation process.

Third, to enhance the robustness of the regression results, we report the estimation outcomes using both TFP_OLS and TFP_FE as dependent variables in all regressions.

Details of these revisions can be found in Section 4: Research design

**References:**

GONG, B. (2018), " Agricultural Reforms and Production in China: Changes in Provincial Production Function and Productivity in 1978-2015", *Journal of Development Economics,* Vol. 13218-31.

LIN, J. Y. (1992), "Rural Reforms and Agricultural Growth in China", *American Economic Review,* Vol. 82 No. 1, pp. 34-51.

2. The capital input is measured using the Perpetual Inventory Method (PIM), but the paper does not specify key details such as the depreciation rate used or how the initial value (base year) was chosen. The authors should provide clarity on these assumptions and perform a sensitivity analysis to ensure the robustness of the capital measurement.

Author's response: As noted in our response to Comment 1, we define the output indicator for agricultural TFP as the total gross output value of agriculture, forestry, animal husbandry, and fisheries in each region (in million RMB), adjusted to constant 2011 prices using the corresponding GDP index. For capital input, we replaced the perpetual inventory method with total mechanical power (in 10,000 kW) as a proxy for fixed capital investment in agricultural production.

3. The paper measures the digital economy using broad indicators like internet penetration and e-commerce, which are comprehensive but may not fully capture the digital transformation in the agricultural sector. The authors should consider including agriculture-specific digital indicators, such as the adoption of digital farming technologies, rural digital infrastructure, and access to digital finance for farmers.

Author's response: We greatly appreciate the reviewer’s insightful suggestion to incorporate agricultural digitalization metrics when evaluating the development of the digital economy. While this is an excellent idea, we attempted to incorporate it by collecting relevant data from China. Unfortunately, these indicators are either unsupported by available data or have limited disclosure periods. For instance, China’s Digital Rural Index has only been published since 2018. Using this data would significantly reduce the sample size for our study. After weighing the pros and cons, we believe our current indicator system represents the most optimal choice given the circumstances. We have also made every effort to evaluate the development of the digital economy comprehensively. To maximize the sample size, we extended the data coverage from 2011–2020 to 2011–2022, further enhancing the credibility and relevance of this study.

4. The paper does not discuss potential biases that might arise when estimating the effect of the digital economy on TFP at the provincial level. It's unclear how the chosen control variables mitigate such biases. The authors should discuss potential biases in greater depth and explain how the chosen control variables (industrial structure, infrastructure, trade dependence, human capital, economic development, urbanization) help address these biases. More agriculture-specific control variables should be considered.

Author's response:：In response to the reviewer’s comment, we made the following revisions: We added additional agriculture-related control variables to further mitigate potential omitted variable bias and enhance the uniqueness of the study. These adjustments also ensure that the model aligns more closely with the paper's core theme. The current set of control variables includes seven factors:

Urbanization (URBAN). The impact of urbanization on agricultural TFP can be seen in two ways. On one hand, it encourages labor migration from agriculture to non-agricultural sectors, driving improvements in production efficiency and resource utilization. Second, urbanization promotes technological diffusion and boosts market demand, thereby incentivizing agricultural innovation. On the other hand, urbanization may cause rural labor outflow and a reduction in arable land, leading to lower agricultural output and TFP. This study measures urbanization using the proportion of urban population to total regional population.

Industrial upgrading (IU). Industrial upgrading reallocates resources from the low-efficiency traditional agricultural sector to more productive modern industries. The growth of the secondary and tertiary sectors also offers increased technological support to agriculture, boosting agricultural TFP. We define industrial upgrading using the following equation (2).

 (2)

The variable xi denotes the share of industry i’s output in total output.

The degree of dependence on foreign trade (OPEN). Import and export trade may influence agricultural TFP in both directions. A higher reliance on foreign trade allows China to benefit from technology spillovers, adopting advanced agricultural technologies and management practices from developed countries. Additionally, international market competition can drive efficiency gains and improve product quality in China’s agricultural sector. However, greater reliance on foreign trade may expose agricultural prices to international market fluctuations, undermining the stability of the sector. Import and export trade may also redirect resources to non-agricultural sectors, crowding out agricultural inputs. We measure foreign trade dependence as the ratio of total imports and exports to GDP, with trade values converted using the annual average exchange rate between the US dollar and the Chinese yuan.

The share of irrigated area (IA). An increase in the share of irrigated area improves water supply to farmland, enhances land use efficiency, and boosts crop yield and quality, thereby positively impacting agricultural TFP. However, excessive expansion of irrigated area may lead to water waste and other environmental issues, ultimately undermining agricultural TFP. We define IA as the ratio of irrigated farmland to total crop area

Rural human capital (HUM). Rural human capital impacts agricultural TFP in two key ways. First, high-quality human capital fosters the adoption and innovation of agricultural technologies, accelerates the spread of modern practices, and boosts productivity. Second, improvements in human capital can facilitate the shift from labor-intensive to knowledge-intensive agriculture, increasing the value added to production. Rural human capital is measured by the average years of education for the population aged 6 and older, as specified in equation (3).

$HUM=\sum_{i=1}^{n} p_{i}y_{i}$ (3)

In equation (3), pi represents the years of education for each level, and n denotes the number of education categories, set to 5. Specifically, we categorize the population's education level into five groups: illiterate or functionally illiterate（$y_{1}=0$）, primary education（$y_{2}=6$）, junior secondary education（$y_{3}=9$）, senior secondary or vocational education（$y_{4}=12$）, and tertiary education or higher（$y_{5}=16$）.

The share of crop disaster-affected area (DISA). A higher proportion of crop disaster-affected area suggests significant damage to arable land, crops, and agricultural infrastructure. This, in turn, negatively affects agricultural output and resource allocation efficiency. We measure DISA as the ratio of affected crop area to total arable land.

Government investment (LGOV). Government investment in agriculture is primarily directed toward infrastructure development, technological research and development, and subsidies for agricultural products. By increasing such investment, production conditions improve, technological progress is accelerated, and agricultural TFP is enhanced. However, it is crucial to note that the effectiveness of government investment is shaped by factors such as policy design, implementation quality, and resource allocation. Over-reliance on direct subsidies or imprecise targeting can result in resource misallocation and incentive distortions in the agricultural sector, ultimately impeding improvements in agricultural TFP. We measure government investment as the expenditure on agriculture, forestry, and water affairs per unit of sown area. Given the high variability of this indicator, we apply a natural logarithm transformation.

As noted in the definition of the control variables, we included four agriculture-related variables and provided a detailed discussion of how each theoretically influences agricultural TFP. This serves as a direct and substantive response to the reviewer’s feedback. The specific revisions and their integration into the manuscript can be found in the main text.

5. While the control variables included are relevant and important for macroeconomic and structural factors, they may not be sufficient for isolating the impact of digital innovation on agricultural TFP. The current controls mainly focus on broader economic indicators rather than agriculture-specific ones. The paper should consider adding control variables that are more specific to agriculture, such as land quality, irrigation access, and farm size. These variables could provide a more accurate reflection of factors affecting TFP in agriculture.

Author's response: This issue is essentially the same as Point 4. The revisions we made in response can be found under “Author's Response” to Point 4.

6. The paper includes a mechanism analysis but lacks agriculture-specific mechanisms. While regional innovation capability is a valuable addition, it would be beneficial to include other variables that directly measure how digital innovations impact agriculture. Consider adding variables like digital infrastructure in rural areas, agricultural R&D, farmer digital literacy, and use of digital finance to provide a more complete understanding of how digital innovations influence TFP.

Author's response: Similar to Issue3, while the reviewer suggested incorporating agriculture-specific mechanisms, we have already included several agricultural variables as controls in the regression equation. More importantly, agriculture-related variables tied to the digital economy are unavailable, making it impossible to use them as mechanism variables. After extensive review of the literature and data, the current approach represents the best possible choice under the circumstances. It is worth noting that in the revised manuscript, we have also updated the data for the two mechanism variables—marketization and regional innovation capacity—through 2022. This further enhances the reliability of our findings.

7. The study could benefit from robustness checks using alternative methods to measure TFP. This would enhance the validity of the results. The authors should consider using multiple methods for TFP measurement and compare the results to ensure robustness.

Author's response: We have already addressed this issue in our response to Comment 1. In line with the reviewer’s suggestion, we report the regression results using both OLS and FE methods to estimate agricultural TFP as the dependent variable in all regression tables. This approach reduces the sensitivity of our findings to a single TFP measurement method, thereby enhancing robustness.

8. The Hansen test yields a high p-value, but the paper does not discuss whether this could indicate overfitting. The authors should address whether the high p-value in the Hansen test suggests potential overfitting and, if so, how this issue was mitigated.

While the paper addresses an important topic, it is not yet ready for publication. The study's main weakness lies in the measurement of TFP and the lack of sufficient discussion on control variables, both of which are critical for ensuring the validity of the findings. I recommend a significant revision of the methodology, with particular attention to the measurement of TFP and the selection of variables, before reconsideration for publication.

Author's response: We appreciate the reviewer’s comment on this issue. In the revised version of the manuscript, we have addressed this concern by adjusting the model specification to avoid a Hansen test p-value of 1 whenever possible. This improvement is reflected in most of our estimation results, with the only exception being Columns (1)–(2) in Table 7, where the Hansen test p-value remains 1. After reviewing the literature and conducting multiple tests on the Stata code, we identified the reason for this outcome. The issue can be summarized as follows: in the "Regional Heterogeneity" section, we divided the sample of China’s provinces into Eastern and Central/Western regions. After updating the sample period to 2022, the Eastern region had 121 observations, creating a disparity with the Central/Western regions. At the same time, we increased the number of control variables from six to seven in order to better examine the impact of the digital economy on agricultural TFP under constant conditions. However, this introduced a "flaw" in the estimation. Specifically, when using the SYS-GMM method, no matter how we adjust the model, the number of instrumental variables exceeds the number of cross-sectional observations in the Eastern region (11), which likely explains the Hansen p-value of 1. Although we could address this by reducing the number of control variables, we found that similar issues with Hansen/Sargan p-values of 1 or very close to 1 have appeared in a number of studies published in top journals（Dennis et al., 2008; Pathan and Faff, 2013）. After careful consideration, we believe that increasing the number of control variables, particularly agricultural controls, is more beneficial for improving the quality of our paper. The detailed revisions can be found in the text, highlighted in blue.

References:

Dennis P. Quinn, A. Maria Toyoda, Does Capital Account Liberalization Lead to Growth?, The Review of Financial Studies, Vol. 21, No. 3, May 2008, pp.1403-1449

PATHAN, S. & FAFF, R. (2013), "Does board structure in banks really affect their performance?", Journal of Banking & Finance, Vol. 37 No. 5, pp. 1573-1589.

9. The paper must make an effort on the formatting. Please make a stronger effort in this regard.

Author's response: Thank you very much for the thorough review. We have revised the article's formatting in accordance with the guidelines of PLOS One.

10. No references were cited in the introduction section. Authors must provide the references.

Author's response: We have made revisions and added the necessary references in the introduction. We present a portion of the content here, with specific revisions detailed in the main text.

Since the initiation of the reform and opening-up policy, China's agricultural sector has undergone rapid development, culminating in a noteworthy total grain output surpassing 695 million tons by the year 2023. This achievement is particularly significant as it has maintained a consistent level exceeding 650 million tons for night consecutive years, attaining an unprecedented pinnacle in historical records. However, concomitant with the progress in China's economic development and the enhancement of people's quality of life, a discernible transformation has transpired in the consumer demand structure for agricultural products [1].

This evolution is characterized by an escalating demand for high-quality agricultural products, juxtaposed against a surplus of low-quality counterparts [2]. Consequently, a pronounced and progressively exacerbating contradiction between supply and demand has emerged at both ends of the agricultural product spectrum [3]. This disjunction underscores the imperative to address the evolving dynamics of consumer preferences and the ensuing challenges faced by the agricultural sector. A nuanced understanding of these shifting patterns is crucial for formulating strategic interventions that can effectively align agricultural production with the contemporary demands of an evolving consumer landscape.

References:

Hu AG, Zhou SJ, R H. Supply-Side Structural Reform: Adapting to and Shaping Ch-ina's New Economic Normal. Journal of Tsinghua University (Philosophy and Social Sciences). 2016; 31(2): 17-22+195.

Qi YD, Liu CH, Ding SL. Digital Economic Development, Employment Structure Op-timization and Employment Quality Upgrading. Economic Perspectives. 2020; (11): 17-35.

Kong XZ. The Basic Connotation and Policy Suggestions on the Structural Reform of Agricultural Supply Side. Reform. 2016; (2): 104-115.

11. Suggestion: Authors should describe all variables used in regression analyses and their sources. They should also describe whether the variables used are ordinal, binary, or other.

Author's response: We have added all variables used in the regression analysis, along with their sources, in both the main text and Table 2, and provided descriptions of their roles.

12. The results are very interesting but lack a discussion with other work in the same field in China and elsewhere.

Author's response: We have added a discussion section at the end of the main text, comparing our work with similar studies from China and other regions or countries in the same field. We present a portion of the content here, with specific revisions detailed in the main text.

Discussion:

(1) The development of the digital economy enhances agricultural TFP

This paper advances research on digital technology and agricultural productivity, offering robust external validity relative to existing studies. The digital economy enhances agricultural TFP in both developing countries, such as China, and advanced economies. Deichmann et al. analyzed the impact of digital technology on agriculture in developing countries [39]. They concluded that digital technology promotes economic inclusivity and boosts agricultural productivity by complementing inputs such as labor and capital. Bocean found that in advanced economies within the European Union, agricultural digitalization strengthened resilience and sustainability while significantly increasing productivity [40]. The digital economy's impact on agricultural TFP is universal, as global digitalization continues to deepen and expand its application in agriculture. Emerging technologies such as the Internet of Things and blockchain will drive the digital economy to further integrate the agricultural value chain, fostering smarter, more automated, and sustainable production. Agricultural producers in both developing and developed countries will increasingly depend on digital technologies to boost productivity, cut costs, and manage market risks. This will further strengthen the digital economy's universal impact on agricultural TFP.

(2) The digital economy's positive impact on agricultural TFP is more pronounced in the central and western regions, as well as in areas with lower TFP

The heterogeneity findings of this study align with existing literature, showing that the digital economy's positive impact on agricultural TFP is stronger in the central and western regions, as well as in areas with lower TFP. Gong examined agricultural productivity trends across China’s regions and found that most regions (23 of 28 provinces) and products (19 of 23) have not achieved convergence. Improvements in irrigation, education, and technology in lagging provinces could help narrow regional productivity gaps [41]. Deng et al. reached similar conclusions, highlighting that regions with less developed agriculture have an advantage in adopting new technologies and translating them into productivity gains [42]. These regions have significant potential to improve efficiency and related indicators through technological advances. In contrast, eastern regions face constraints, such as limited land resources, that hinder further marginal gains. This finding offers new empirical evidence for studies in developing countries like China, though it may not align with trends observed in some developed countries. Plastina et al. examined the impact of technological progress on agricultural TFP in the United States [43]. They found the strongest positive effect in the central region, followed by the western region, with the weakest effect in the east. This pattern reflects the high overall level of agricultural development and the more balanced spatial distribution in developed countries like the U.S. The central region, as the primary grain-producing area with commodity crop agriculture, benefits most from advanced technologies, while the eastern and western regions, which focus on dairy farming and specialty agriculture, experience a smaller impact.

(3) The digital economy impacts agricultural TFP by boosting regional innovation and fostering marketization

The development of information technology broadens farmers' access to information, offering more learning and training opportunities that enhance their production skills. This finding is supported by studies such as Deichmann et al. [39]. Additionally, other scholars have shown that it promotes human capital accumulation, which in turn drives overall agricultural productivity [44]. The findings in the mechanism section — that the digital economy boosts agricultural TFP by enhancing regional innovation and promoting marketization—extend prior research with a more general conclusion. On one hand, the digital economy enhances data-driven precision in information matching and market responses, fostering innovative business models such as agricultural e-commerce platforms, crowdfunding for agricultural products, and the agricultural sharing economy. This, in turn, boosts agricultural TFP. This finding expands on the role of information technology in broadening farmers' access to information, reducing information asymmetry, and facilitating marketization. On the other hand, the digital economy fosters an innovative ecosystem that promotes collaboration among various stakeholders, optimizes the allocation of innovation resources, and generates knowledge spillovers. This supports regional economic transformation and sustainable development, going beyond mere human capital accumulation to enhance regional progress across multiple dimensions. Thus, it offers a more comprehensive view of the digital economy's role in promoting human capital aggregation.

References:

39.	Deichmann U, Goyal A, Mishra D. Will digital technologies transform agriculture in developing countries? Agricultural Economics. 2016; 47(1): 21-33. https://doi.org/10.1111/agec.12300

40.	Bocean C. G. A Cross-Sectional Analysis of the Relationship between Digital Techno-logy Use and Agricultural Productivity in EU Countries. Agriculture, 14(4), 519. https://doi.org/10.3390/agriculture14040519

41.	Gong B. Agricultural productivity convergence in China. China Economic Review. 2020; 60: 101423. https://doi.org/10.1016/j.chieco.2020.101423

42.	Deng H, Jin Y, Pray C, et al. Impact of public research and development and extens-ion on agricultural productivity in China from 1990 to 2013. China Economic Review. 2021; 70: 101699. https://doi.org/10.1016/j.chieco.2021.101699

43.	Plastina A, Lence H. S, Ortiz-Bobea A. How Weather Affects the Decomposition of Total Factor Productivity in U.S. Agriculture. Agricultural Economics. 2021; 52: 215-234. https://doi.org/10.1111/agec.12615

44.	Zhou XX, Chen T, Zhang BB. Research on the Impact of Digital Agriculture Develo-pment on Agricultural Green Total Factor Productivity. Land. 2023; 12(1): 195. https://doi.org/10.3390/land12010195
